# Nanostructured Pd-Based Electrocatalyst and Membrane Electrode Assembly Behavior in a Passive Direct Glycerol Fuel Cell

**DOI:** 10.1186/s11671-019-2871-8

**Published:** 2019-02-11

**Authors:** N. Yahya, S. K. Kamarudin, N. A. Karim, S. Basri, A. M. Zanoodin

**Affiliations:** 1 Fuel Cell Institute, Universiti Kebangsaan Malaysia, UKM, 43600 Bangi, Selangor Malaysia; 2 Department of Chemical and Process Engineering, Universiti Kebangsaan Malaysia, UKM, 43600 Bangi, Selangor Malaysia; 3 Malaysian Institute of Chemical and Bioengineering Technology, Universiti Kuala Lumpur, Melaka, Malaysia

**Keywords:** Palladium, Catalyst, Membrane, Carbon nanofiber, Glycerol oxidation

## Abstract

The aim of this study was to synthesize, characterize, and observe the catalytic activity of Pd_1_Au_1_ supported by vapor-grown carbon nanofiber (VGCNF) anode catalyst prepared via the chemical reduction method. The formation of the single-phase compounds was confirmed by X-ray diffraction (XRD) and Rietveld refinement analysis, which showed single peaks corresponding to the (111) plane of the cubic crystal structure. Further analysis was carried out by field emission scanning emission microscopy (FESEM), energy dispersive X-ray analysis (EDX), nitrogen adsorption/desorption measurements, and X-ray photoelectron spectroscopy (XPS). The electrochemical performance was examined by cyclic voltammetry tests. The presence of mesoporous VGCNF as support enables the use of a relatively small amount of metal catalyst that still produces an excellent current density (66.33 mA cm^−2^). Furthermore, the assessment of the kinetic activity of the nanocatalyst using the Tafel plot suggests that Pd_1_Au_1_/VGCNF exerts a strong electrocatalytic effect in glycerol oxidation reactions. The engineering challenges are apparent from the fact that the application of the homemade anode catalyst to the passive direct glycerol fuel cell shows the power density of only 3.9 mW cm^−2^. To understand the low performance, FESEM observation of the membrane electrode assembly (MEA) was carried out, examining several morphological defects that play a crucial role and affect the performance of the direct glycerol fuel cell.

## Introduction

Glycerol might exceed demand as it is formed as a by-product from biodiesel production [1]. However, the good properties of glycerol which are high-energy density with three hydroxyl group, non-toxic, and non-flammable make it emerge as a worthy chemical that can be converted into value-added products. After pretreatment, for instance, using electrodialysis [2], glycerol can be applied as fuel to fuel cells [3]. Fuel cells are among the most efficient ways to harvest energy from glycerol oxidation with various types of anode materials and also catalysts.

Palladium began to receive attention and fame among researchers worldwide in 2010, when Richard Heck, Ei-ichi Negishi, and Akira Suzuki received the Nobel Prize in recognition of their success in the discovery and development of palladium-catalyzed carbon-carbon bond formation [4]. Their study had a significant impact in the field of catalysis. Furthermore, it has become a source of inspiration for researchers to apply palladium metal catalysts in various areas ranging from industrial materials and pharmaceuticals to the harnessing of energy through fuel cells. Palladium is one of the metals in the platinum group. The similar chemical properties with platinum make palladium a very appropriate match for replacing the more expensive platinum [5, 6]. Palladium has a unique electronic configuration with the 4*d*^10^5*s*^0^ ground-state electronic configuration, making it the only transition metal that combines a filled *d* orbital with an empty frontier *s* orbital [7]. The unique properties of palladium appear to indicate the ability of palladium to catalyze the oxidation of olefins [8].

However, when used alone as a catalyst, palladium (Pd) shows a lack of stability, as well as high rates of deactivation and deterioration [9, 10]. Many studies have shown that a mixture or combination of Pd and Au metals can be used in a number of applications. The addition of Au into Pd is believed to greatly enhance the catalytic activity, stability, and selectivity, as well as provide resistance to poisoning. Au has a higher electronegativity, and thus, the charge is transferred from Pd to Au, making Au as a promoter that plays the role of isolating the Pd monomer site, thereby preventing the occurrence adsorption of CO by promoting further reaction mechanism on another pathway [11, 12]. Furthermore, the alloying of these two metals leads to the gain of *s* and *p* electrons and the loss of *d* electrons for Au, whereas Pd loses *s* and *p* electrons but gains *d* electrons [13]. The *d*-character is much more important than *s*, *p*-character for transition metal like Pd and Au, which allows the formation of chemisorption bonds and improves catalytic properties.

The electrocatalytic activity of the bimetallic Pd structure is better than that of the monometallic structure, in which the presence of oxophilic metals creates a tendency to form oxides by hydrolysis or abstraction of oxygen, often from organic compounds that assist in glycerol oxidation [14]. Referring to the bifunctional theory of electrocatalysis, the electrooxidation of primary alcohol (e.g, methanol, ethanol, and glycerol) to CO_2_ and R–COOH (or ${\mathrm{CO}}_3^{2-}$ and R − COO^−^ in alkaline medium) requires the activation of H_2_O under OH-adsorbed species (OHads) at the catalyst’s surface. Thus, it will provide the extra oxygen atom and assist in the complete alcohol oxidation reaction, according to the following chemical reaction occurring in alkaline electrolyte:

1 $$ \mathrm{Pd}+\mathrm{RCH}2\mathrm{OH}\to \mathrm{Pd}-{\left(\mathrm{R}{\mathrm{CH}}_2\mathrm{OH}\right)}_{\mathrm{ads}} $$

2 $$ \mathrm{Pd}-{\left({\mathrm{RCH}}_2\mathrm{O}\mathrm{H}\right)}_{\mathrm{ads}}+3{\mathrm{OH}}^{-}\to \mathrm{Pd}-{\left(\mathrm{RCO}\right)}_{\mathrm{ads}}+3\mathrm{H}2\mathrm{O}+3{\mathrm{e}}^{-} $$

3 $$ \mathrm{Pd}-{\mathrm{OH}}^{-}\to \mathrm{Pd}-{\mathrm{OH}}_{\mathrm{ads}}^{-}+{\mathrm{e}}^{-} $$

4 $$ \mathrm{Pd}-{\left(\mathrm{RCO}\right)}_{\mathrm{ads}}+\mathrm{Pd}-{\mathrm{OH}}_{\mathrm{ads}}^{-}\to \mathrm{Pd}-\left(\mathrm{RCO}\mathrm{OH}\right)+\mathrm{Pd}\ \mathrm{rds} $$

5 $$ \mathrm{RCOOH}+\mathrm{OH}\to {\mathrm{RCOO}}^{-}+\mathrm{H}2\mathrm{O} $$

The fast dissociative adsorption of alcohol on the catalyst surface can be related to Eq. (1) and Eq. (2). The rate-determining step in Eq. (4) means that the alcohol oxidation reaction strongly depends on the coverage of the adsorbed acyl and hydroxyl species, RCOads and OHads, on the surface of the nanocatalyst, generally leading to the formation of acetate [15].

Palladium alloys can be worked to improve hardness and strength without detriment to the corrosion resistance of the other metals [16]. Pd–Au bimetallic catalyst is a good coupling and profoundly active nanoparticle catalyst. Yan et.al [17] and Hu et.al [11] observe that Pd nanoparticles are a highly active catalyst, but they are not stable, and the catalytic activity drops due to decomposition of PdO. Zhang et al. [18] conducted another interesting study on the incorporation of Pd into carbide, which resulted in a peak current value for Pd/Mo_2_C of 1000 mA mg^−1^, a value that is two times greater than that of Pd/C (498 mA mg^−1^); this result reveals alloy catalyst proved to give a stable and good poisoning-resistant electrocatalyst for the oxidation of glycerol in alkaline media. Ferreira et al. also [19] conducted a study on pH factors in the electrooxidation of glycerol on palladium–rhodium electrodeposits in alkaline media. Authors showed that the palladium–rhodium surfaces present suitable catalytic activity towards the electrooxidation of glycerol and suggested that the presence of rhodium helps to improve the low ability of palladium to cleave C–C bonds in strongly alkaline media. Rezaei et al. [20] reported that Pd/Cu/nanoporous stainless steel electrodes for the oxidation of glycerol in the presence of Cu resulted in enhanced electrocatalytic activity and stability of the porous Pd film for glycerol oxidation reactions. Another interesting study by Geraldes et al. [21] showed that the combination of three metal PdAuSn/C catalysts not only promoted the dissociative activity of glycerol molecules during the oxidation process but also resulted in higher durability, stability, and power density.

However, there is an ongoing debate regarding the best strategies for reducing the use of metal catalyst. In the present study, a carbon material, namely the vapor-grown carbon nanofiber (VGCNF), has been introduced as catalyst support to minimize the PdAu loading for the electrooxidation of glycerol in fuel cell applications. Despite a growing body of research on the PdAu alloy, no studies have been performed for the use of VGCNF as catalyst support. VGCNF has been used as catalyst support because of its diverse characteristics of highly graphitic carbon with excellent thermal and electrical conductivities [22]. In addition, VGCNF can be found in large quantities at an affordable price [23]. The primary goal of this paper is to synthesize and characterize Pd_1_Au_1_/VGCNF to contribute to this growing area of research by exploring the utilization of this homemade catalyst in the direct glycerol fuel cell (DFGC).

Catalysts were synthesized and then applied to the passive direct glycerol fuel cell (DGFC). Catalysts in the electrodes of the DGFC serve a critical function in reactions used to generate electricity through the conversion of chemical fuel to electrical energy. The application of Pd_1_Au_1_/VGCNF in passive DGFC to power low-watt mobile electronic devices has never been studied previously. The passive mode in DGFC means that fuel is supplied from the reservoir to the anode compartment without a fuel pump, while at the cathode side, the oxygen diffuses passively from the ambient air [24]. The DGFC is operated in the passive mode, and there are no auxiliary devices that are used for operational purposes. The glycerol flow rates are difficult to control, and the intermediate species during glycerol oxidation limit the activity of the catalyst and contribute to the catalyst decay leading to the lower performance of a passive DMFC compared to an active DMFC.

Many challenges must be faced for the commercialization of passive fuel cells. In many studies, the synthesized catalyst performed well in the half-cell testing via cyclic voltammetry. However, the subsequent applications of the catalyst in passive fuel cells did not show a very encouraging power density. Therefore, this study will also thoroughly discuss several factors such as the structural catalyst layer, MEA manufacturing issues, and AEM stability that contribute to the low performance of the fuel cell.

## Materials and Methods

### Chemicals

All precursor metal salts and chemical reagents such as gold(III) chloride trihydrate (HAuCl_4_·3H_2_O), palladium chloride (PdCl_2_), trisodium citrate (Na_3_Ct), sodium borohydride (NaBH_4_), vapor-grown carbon nanofiber (VGCNF), sodium hydroxide, glycerine, 2-propanol, and 5 wt% Nafion solution were purchased from Sigma-Aldrich (USA).

### Instrumentation

For the physical analyses of electrocatalysts, techniques such as X-ray diffraction (XRD), field emission scanning electron microscopy (FESEM), energy-dispersive X-ray (EDX) spectroscopy, and surface area analysis by BET were used to examine the electrocatalyst structure, morphology, elemental composition, crystallite size, and porosity of the sample. XRD is used for phase identification in crystalline materials. The instrument used in this work is a Bruker D8 Advance diffractometer equipped using a Cu Ka source (*λ*= 1.54056 Å). The diffractograms were recorded at 2q in the range 20 to 90^°^, with a step size of 0.05 and scan time of 2 s per step. The Scherrer equation is used to determine the size of the crystal particles in the powder as follows:

$$ \beta =\frac{K\lambda}{L\cos \theta } $$

where *λ* is the X-ray wavelength, *θ* is the Bragg reflection angle, and $K=2\sqrt{\left(\mathit{\ln}2\right)/}\left(\pi \right)$ is the constant. Furthermore, Rietveld refinement analysis of X-ray powder diffraction data was conducted using the PANalytical X’Pert HighScore software [25]. In the Rietveld analysis, the experimental profiles were fitted with the most suitable pseudo-Voigt analytical function [26]. The minimization of the difference between the observed and simulated powder diffraction patterns was carried out using the reliability index parameter such as the residuals *R*_wp_ for the weighted residual pattern, *R*_exp_ for the evaluation of the data quality or the expected error, and goodness of fit (GOF) which typically summarizes the quality of the data by comparing *R*_wp_ to *R*_exp_. The value of GOF equal to 1 or below 4 means the model is as good as possible [27]. The accuracy and quality of the fit of the calculated to the experimental diffraction data can be determined through all these parameters and are given by the following:

6 $$ {R}_{\mathrm{wp}}=\left[\frac{\sum {w}_{\mathrm{i}}{\left({y}_{\mathrm{i}\mathrm{o}-}{y}_{\mathrm{i}\mathrm{c}}\right)}^2}{\sum {w}_{\mathrm{i}}{y}_{\mathrm{i}\mathrm{o}}^2}\right] $$

7 $$ {R}_{\mathrm{exp}}={\left[\frac{N-P}{\sum {w}_{\mathrm{i}-}{y}_{\mathrm{i}\mathrm{o}}}\right]}^{1/2} $$

8 $$ \mathrm{GOF}={X}^2={\left[\frac{R_{\mathrm{wp}}}{R_{\mathrm{exp}}}\right]}^2 $$

where *y*_i(o)_ are the experimental intensities, *y*_i (cal)_ are the calculated intensities, *w*_i_ = (1/*y*_i(o)_) is the weight of the experimental observation, *N* is the number of experimental observations, and *P* is the number of fitting parameters.

The topographical and elemental information of the nanostructured catalyst were obtained using a Gemini SEM 500 field emission scanning electron microscope equipped with an energy-dispersive X-ray spectrometer that can provide three-dimensional images and information on the elemental composition of the sample under analysis. Nitrogen adsorption/desorption isotherms were used to evaluate the surface properties of the synthesized powder catalyst Pd_1_Au_1_/VCGNF such as Brunauer–Emmett–Teller (BET) surface area. Meanwhile, the pore size distribution was calculated from the adsorption branches of the isotherms (for P/P0 > 0.35) using the Barret–Joiner–Halenda (BJH) method.

### Catalyst Synthesis

The approach used to synthesize the electrocatalyst used in this study is based on the chemical reduction method [28]. This is the simplest method which allowed the formation of the Pd_1_Au_1_ bimetallic alloy supported with vapor-grown carbon nanofiber (VGCNF). The electrocatalyst synthesis started with 2 ml PdCl_2_ (0.05 M) mixed with 7 ml gold(III) chloride trihydrate (HAuCl_4_·3H_2_O) (0.012 M). The mixed solution was added dropwise to some amount of trisodium citrate (0.5 M).

Trisodium citrate acts as a stabilizing agent to control the aggregation of the nanoparticles by lowering the surface tension between the solid particles and the solvent [29]. Subsequently, the above mixed solution was added dropwise to the stirred VGCNF slurry (isopropanol + DI water) and was stirred for 3 h. The reduction of the metal precursors was carried out using an excess amount of freshly prepared ice-cold (0.5 M) sodium borohydride (NaBH_4_), and the solution was stirred overnight. A longer reaction time will allow sodium borohydride with its strong reducing abilities to react with the products. The molar ratio of NaBH_4_ to metal ions is 5 to 15, which provides a better catalyst dispersion and surface composition of the Pd_1_Au_1_ bimetallic alloy nanoparticles. The solution was kept under magnetic stirring overnight, filtered and washed with DI water several times to completely remove solvent, and was dried at 80 °C for 10 h.

### Electrochemical Analysis

Cyclic voltammetry experiments were performed for the electrochemical analysis of the electrocatalyst. The cyclic voltammetry measurements were performed using an Autolab (PGSTAT101) electrochemical workstation at room temperature. The catalyst ink was prepared by dissolving 5 mg of electrocatalyst in a mixture of 150 μl distilled water, 150 μl isopropyl alcohol, and 50 μl 5 wt% Nafion®. To ensure the mixture is well mixed, the sample undergoes ultrasonication for 30 min. The 2.5 μl aliquot of the electrocatalyst ink was deposited on the glassy carbon electrode by using a micropipette and was then left to dry at room temperature. The reference electrode was Ag/AgCl (in 3 M KCl^−1^), and the counter electrode was a Pt. The electrochemical characterization of the electrocatalysts was performed by a cyclic voltammetry (CV) test in the potential range of − 0.7 V to 0.4 V and at the scan rate of 50 mVs^−1^ in a 0.5 M glycerol + 0.5 M NaOH solution. Both solutions were deoxygenated by bubbling with N_2_ at 200 ml min^−1^ for 30 min prior to performing any measurement for the glycerol oxidation reaction.

### Electrochemical Impedance Spectroscopy (EIS)

EIS of the running FC was performed using a frequency response analyzer (FRA) Solartron 1260, directly connected to the electronic load (RBL488-50-150-800). EIS was performed in galvanostatic mode using an AC signal of amplitude 200 mA. Impedance spectra were collected by sweeping frequencies between 100 kHz and 0.005 Hz. The amplitude of the sinusoidal potential signal was 10 mV. Each scan contained about 100 data points. Five full spectra for each current density value were acquired, and the impedance spectrum finally used in the discussion was the result of an averaging procedure.

### Preparation of Sodium-Doped Anion Exchange Membrane

The treatment of the anion exchange membrane (AEM) was carried out by immersing the AEM in a 1.0 M sodium hydroxide aqueous solution for 1 h. This process was repeated three times, with replacement of the NaOH solution by a fresh NaOH solution to obtain an excess of the desired ion.

### Membrane Electrode Assembly

The membrane electrode assembly (MEA) had an active area of 2.0 × 2.0 cm and consisted of two single-sided electrodes and an anion exchange membrane. An Electrochem, Inc. carbon cloth (CC-060) was used as the substrate layers (DLs) of the fuel cell electrodes. This is mainly due to its high porosity and good electrical conductivity. Microporous layers consisting of Vulcan XC72 carbon powder were mixed with a hydrophobic agent (20 wt% PTFE) in suspension in isopropanol and were carefully cast onto a carbon cloth to form a gas diffusion layer to obtain the loading of 3.5–4 mg cm^−2^. After drying, P_1_Au_1_/VGCNF catalyst ink mixed with 15 wt% PTFE in isopropanol was deposited on the GDL to obtain the anode electrodes. Meanwhile, Pt/C serves as cathode electrodes catalyst containing an ionomer solution (Fumatech Fumion® FAA-3 ionomer) 5 wt% in isopropanol. The catalyst loadings in the anode and cathode were 1.0 mg cm^−2^. Eventually, the MEA was formed by sandwiching the membrane between the two electrodes via hot pressing at the pressure of 280 kg/cm^2^ and temperature of 100 °C for 3 min.

### Performance Evaluation of Passive Alkaline DGFC

The performance of the MEA was evaluated through I-V curve measurements by using a homemade single cell using Autolab (PGSTAT101). The reservoir with a volume of 8 ml was attached to the one side of the anode end plate. Fuel feeding or injection was done through three small holes on the upper side of the anode plates. The cell was assembled horizontally with the anode side on the top and the cathode facing down to the ambient air. Stainless steel, which has a very good conductivity, was applied as the carbon collector. The prepared MEAs were located between the two carbon collectors. Both electrodes have the geometric surface area of 4 cm^2^. Eventually, eight stainless steel nut-and-bolt pairs were employed to hold all components together to form a single cell. Each measurement was started after the cell activation for 2 hours. The cell voltage was recorded after setting up the current for 3 min to stabilize the voltage. The passive DMFC was operated at ambient temperature.

## Results and Discussion

### Electrocatalyst Physical Characterization

**Fig. 1:**
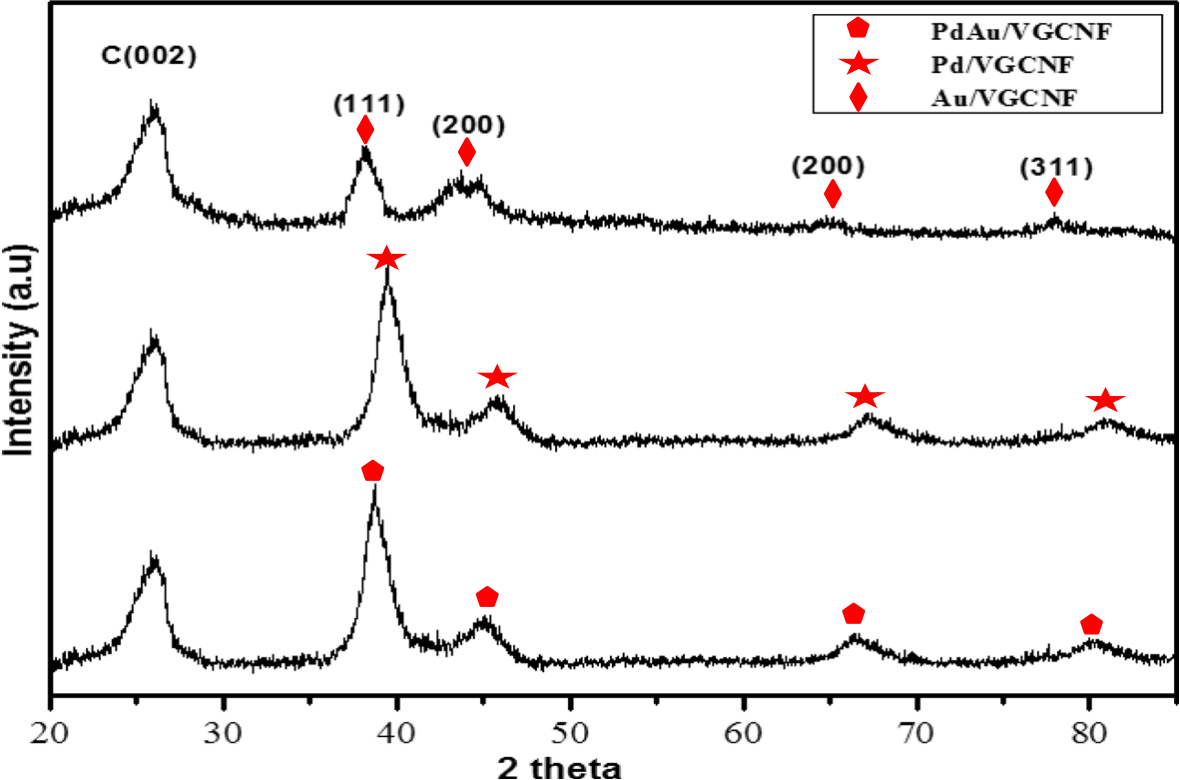
Diffractograms of the different electrocatalysts Pd_1_Au_1_/VGCNF, Pd/VGCNF, and Au/VGCNF

The X-ray diffraction patterns for the three synthesized electrocatalyst samples which are Au/VGCNF, Pd/VGCNF, and Pd_1_Au_1_/VGCNF are shown in Fig. 1, while the analysis data are compiled in Table 1. The X-ray diffraction pattern of the electrocatalysts shows a cubic structure (Fm-3m space group) without a second phase. The first diffractogram peak at 26.0° can be assigned to graphite-structured carbons (002) of the raw VGCNF diffraction planes of hexagonal graphite (JCPDS card file 41-1487) (Rajarao and Bhat [30]). Each diffractogram pattern exhibited has four diffraction peaks that can be indexed to diffraction from the plane (1 1 1), (2 0 0), (2 2 0), and (3 1 1) fcc.

**Table 1:** Physicochemical parameters of electrocatalysts by BET analysis

Electrocatalysts	*S*_BET_ (m^2^/g)	*V*_volume pores_ (cm^3^/g)	*D*_p_ (nm)	Types of pores
VGCNF	30.56	0.415	4.0	Meso
Au/VGCNF	26.33	0.260	3.8	Meso
Pd/VGCNF	26.36	0.264	3.7	Meso
Pd_1_Au_1_/VGCNF	27.32	0.333	3.6	Meso

Figure 1 shows the diffraction peak of the Au/VGCNF electrocatalyst samples at around 38.3 °C, 44.5 °C, 64.7 °C and 77.7 °C and can be indexed to crystallographic planes (1 1 1), (2 0 0), (2 2 0), and (3 1 1), respectively, which can be classified as the Fm-3m (JCPDS No. 65-8601, a = b = c = 0.4072 nm). Figure 1 shows the diffraction pattern of Pd/VGCNF electrocatalyst sample around the 2θ values of 40.12 °C, 46.66 °C, 68.12 °C, and 82.10 °C which corresponds to the following planes (1 1 1), (2 0 0), (2 2 0), and (3 1 1) (JCPDS No. 05-0681, a = b = c = 0.3889 nm). In Fig. 1, for PdAu bimetallic sample, Pd and Au appear to share a single diffraction peak indicating that homogeneous alloy formation has occurred.

**Fig. 2:**
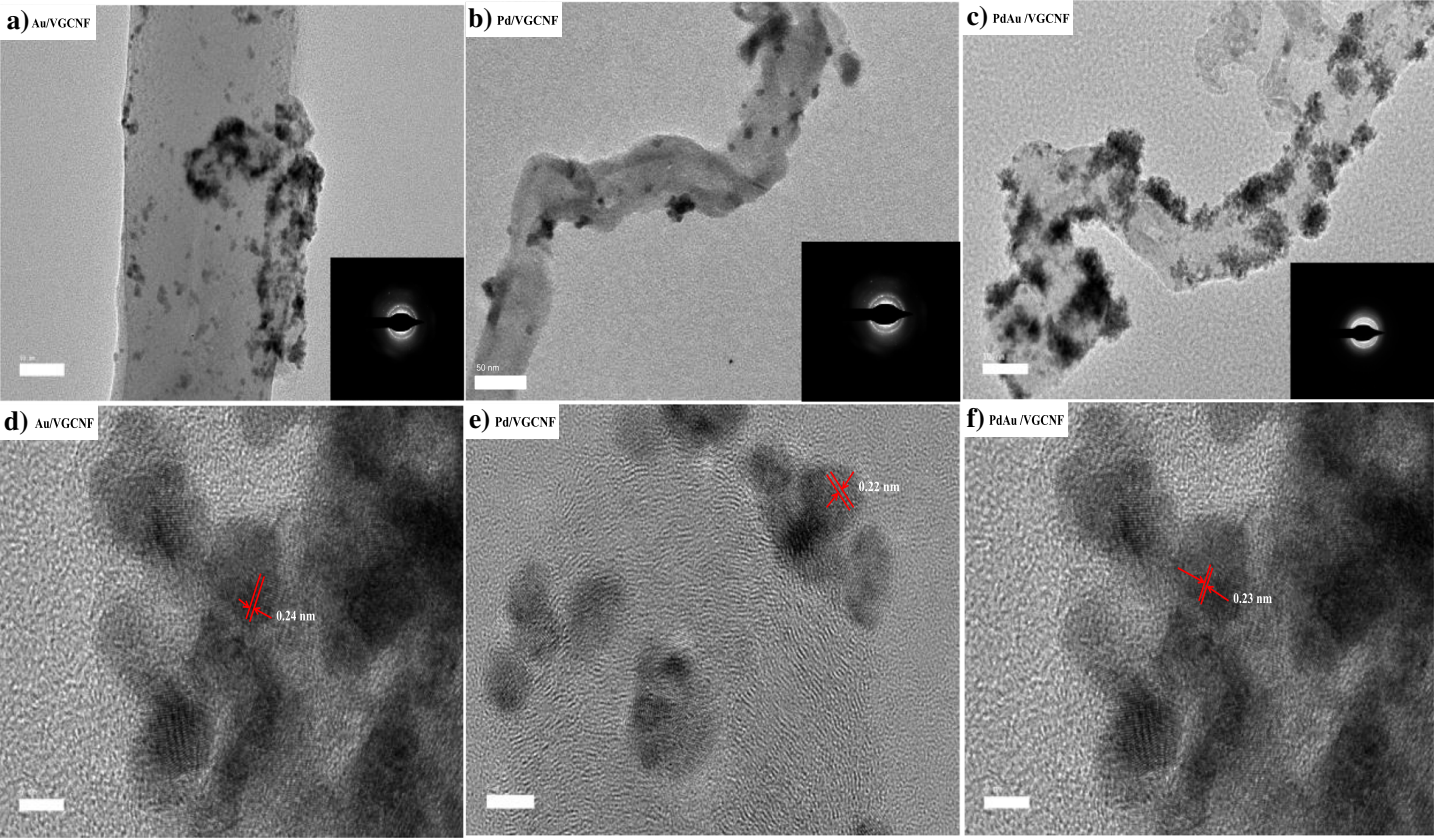
**a**–**c** Typical images of TEM and SAED pattern for Au/VGCNF, Pd/VGCNF, and Pd_1_Au_1_/VGCNF. **d**–**f** HRTEM images for Au/VGCNF, Pd/VGCNF, and Pd_1_Au_1_/VGCNF electrocatalysts

Typical PdAu nanoparticles supported on VGCNF were basically fabricated by the co-reduction of the metal precursors (i.e., ${\mathrm{AuCl}}_4^{-}$ and ${\mathrm{PdCl}}_4^{2-}$) in the presence of sodium borohydride as reduction agent. Figure 2a–c shows the typical TEM image of the Au/VGCNF, Pd/VGCNF, and PdAu/VGCNF electrocatalysts. It can be seen that Au, Pd, and PdAu nanoparticle morphology display a moderate degree of agglomeration behavior with the homogenous distribution on VGCNF. The crystal structure pattern was analyzed by selected area electron diffraction (SAED) analysis (inserted in Fig. 2a–c) and indicates that Au/VGCNF, Pd/VGCNF, and PdAu/VGCNF are typically fcc polycrystalline structure. The high-resolution TEM (HRTEM) image observed in Fig. 2d, e indicates a spacing distance of 0.24 nm and 0.22 nm which corresponds to the interplanar distance of the (111) plane for Au and Pd, respectively. Figure 2f revealed the *d*-spacing values of the lattice fringes determined from the marked regions are 0.230 nm, corresponding to the (111) planes of the face-centered cubic (fcc) PdAu alloy.

**Fig. 3:**
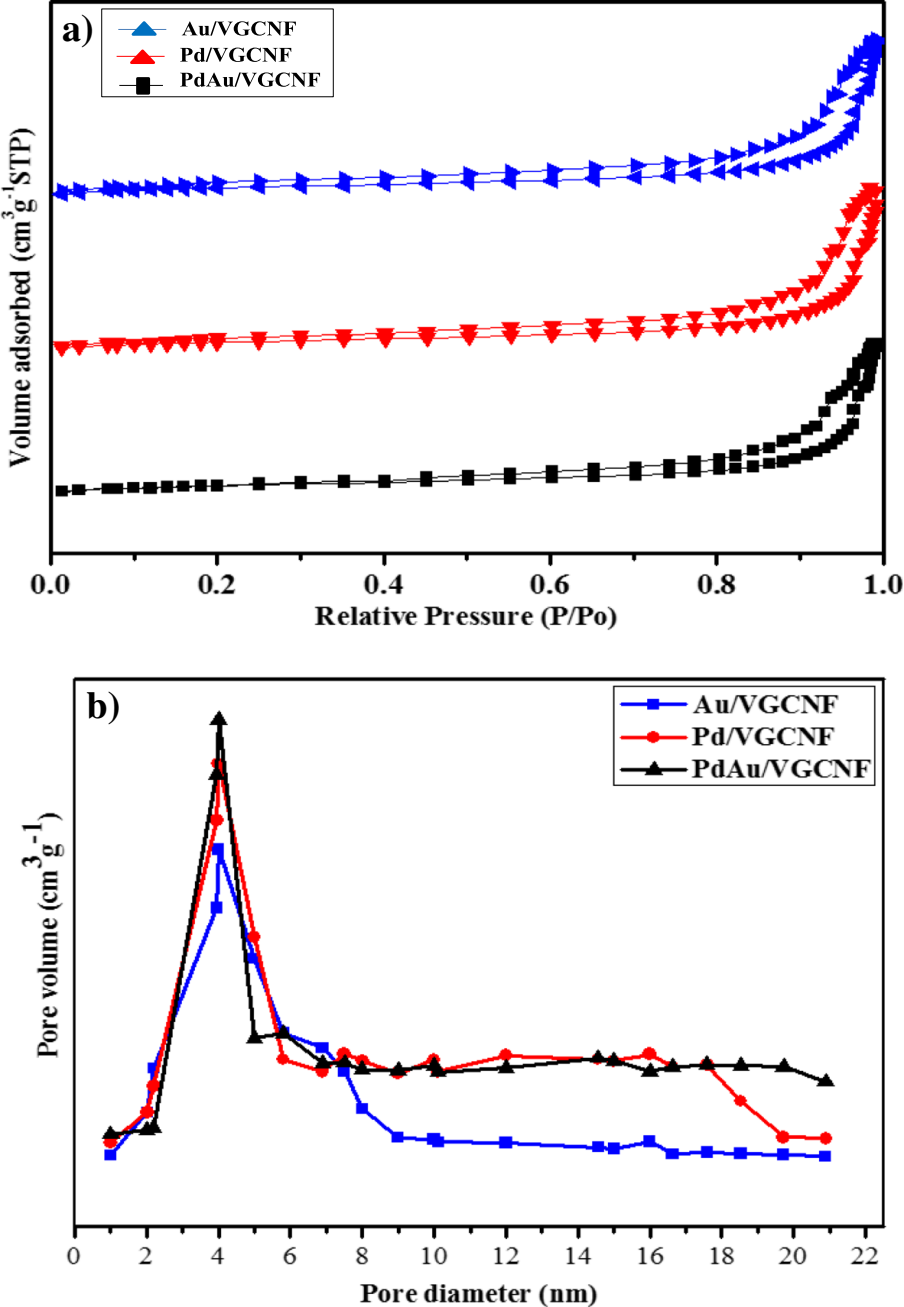
**a** N_2_ adsorption/desorption isotherms of Au/VGCNF, Pd/VGCNF, and Pd_1_Au_1_/VGCNF. **b** Pore size distribution curve of Au/VGCNF, Pd/VGCNF, and Pd_1_Au_1_/VGCNF

The BET surface area and porosity analysis are effective tools for the evaluation of the capability of Au/VGCNF, Pd/VGCNF, and Pd_1_Au_1_/VCGNF to act as an adsorbent. BET revealed the amount of the adsorbed material (adsorbate) and its adsorption degree onto the adsorbent surface on the solid (the adsorbent) [31]. The BET surface area of the Au/VGCNF, Pd/VGCNF, and Pd_1_Au_1_/VGCNF sample was analyzed using nitrogen adsorption/desorption isotherms as shown in Fig. 3a. The isotherm curve reflects the isotherm shape and will provide a qualitative assessment of the porosity of the materials. In this study, the observed isotherm shape indicates that Au/VGCNF, Pd/VGCNF, and Pd_1_Au_1_/VCGNF are found to be type IV (based on IUPAC classification) and shows steep slopes in the relative pressure range of 0.83–0.99 P/P_0_, which is characteristic of an existence of a mesoporous material [32]. Materials with mesoporous structures show good morphological stability and active catalysts due to an increased volume of adsorption, thereby enhancing catalytic activity.

Surface area, pore volume, and pore diameter value of the electrocatalyst are presented in Table 1. From the table, VGCNF has a surface area of 30.56 m^2^/g, the pore volume of 0.415 cm^3^/g, and the pore diameter of 7.0 nm. The presence of metal catalysts (Au, Pd, and PdAu) above the catalyst support was found to decrease surface area, pore volume, and pore diameter. This is due to the catalyst metal which has been scattered and meets the surface of the catalyst support. Even after the catalyst metal is scattered on VGCNF, the H1 type hysteresis loop (P/Po 0.83–0.99) indicates the mesopore structure of the material is unaffected. It can be concluded that the catalyst metal has been well spread on the surface of VGCNF and in line with the TEM image indicating that the catalyst metal is scattered uniformly on the surface of the catalyst support.

As seen from the BJH pore size distribution of Au/VGCNF, Pd/VGCNF, and Pd_1_Au_1_/VCGNF presented in Fig. 3a, it shows a narrow pore diameter or pore diameter at around 4 nm (pore radius), while the pore size range observed in all catalysts are located between 2 and 5 nm, which makes the material a unique adsorbing medium [31]. The Au/VGCNF, Pd/VGCNF, and Pd_1_Au_1_/VGCNF electrocatalysts have small pore volume compared to VGCNF, owing to some porous space inside the support media being blocked with metal catalyst nanoparticles or other chemical reactions during the process of nanoparticle decoration and consequently resulting in the drop of the pore volume. However, the BET surface area reported does not refer to the entire electrocatalytic surface area. Therefore, the electrochemical analysis needs to be carried out in details to obtain active surface area.

**Fig. 4:**
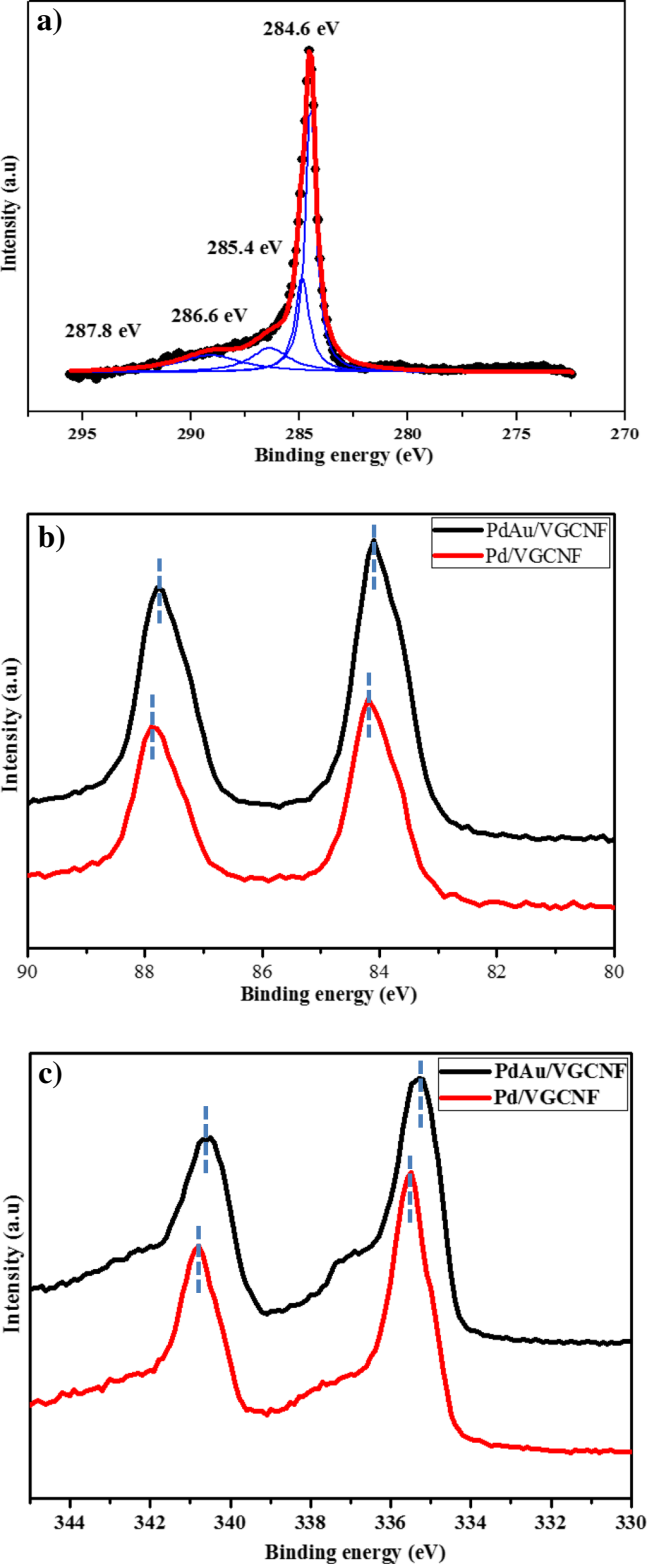
High-resolution XPS spectra of Pd/VGCNF, Au/VGCNF, and Pd_1_Au_1_/VGCNF. **a** Pd 4*f*_5/2_ and Pd 4*f*_7/2_. **b** Au 4*f*_5/2_ and Au 4*f*_7/2_ and **c** C 1*s*

XPS analysis was conducted to investigate C 1*s*, Pd 3*d*, and Au 4*f* in Au/VGCNF, Pd/VGCNF, and PdAu/VGCNF electrocatalysts samples. The high-resolution peak spectrums of the C 1*s* peak are shown in Fig. 4a; four components, namely 284.6 eV (C-C), 285.4 eV (C-O-C), 286.6 eV (C-OH), and 287.8 eV (C = O), could be observed which determine the chemical structure of VGCNF in the electrocatalyst samples. XPS was employed to characterize the chemical, electronic state, and surface composition of the Au/VGCNF, Pd/VGCNF, and PdAu/VGCNF. Table 2 shows the weight percentage and binding energy for Pd/VGCNF, Au/VGCNF, and Pd_1_Au_1_/VGCNF. Figure 4b depicts the regional Au 4*f* spectra of the Au/C and PdAu/C. In the Au 4*f* spectrum of the Au/VGCNF, double peaks contain a low-energy band (Au 4*f*_7/2_) and high-energy band (Au 4*f*_5/2_) at 84.1 and 87.7 eV, which confirm the reduction of ${\mathrm{AuCl}}_4^{2-}$ to Au^0^. In Fig. 4c for Pd/VGCNF, the peak located at 335.4(Pd 3*d*_5/2_) and 340.7 eV (Pd 3*d*_3/2_) are attributed to Pd 3*d* which confirm the reduction of ${\mathrm{PdCl}}_4^{2-}$ to Pd^0^. Meanwhile, for bimetallic PdAu/VGCNF, it can be observed that both binding energy of Au 4*f* and Pd 3*d* slightly deviate to lower angle compared to Au/VGCNF and Pd/VGCNF. The shift of the binding energies provides strong justification of the electron transfer from Pd to Au, which possibly can be related to the perturbed electronic interaction between Pd and Au atomic orbit and further verifying the formation of the alloy nanostructures. The amount of Pd and Au can be determined through the peak intensities and shows that the Pd to Au ratio is 79:21, which is much higher than the calculated value (50:50). This means that Pd atom was enriched in the outer part of the catalyst surface. This occurs owing to the difference in the reduction rates of Au(III) and Pd(II). It can be assumed that Au nuclei formed first, followed by the addition of Pd atoms onto the nuclei, and leading to the formation of alloy nanostructures with Pd-enriched surfaces. This observation confirms the existence of metallic Pd and Au in the outer surface of the Pd_1_Au_1_/VCGNF nanocatalyst sample.

**Table 2:** Quantitative results carbon (C), palladium (Pd), and aurum (Au) elements on the surface of the electrocatalysts and binding energy from XPS analysis

Electrocatalysts	Weight percentage (%)			Atomic percentage (%)			Peak binding energy (eV)	Peak binding energy (eV)
	C	Pd	Pd_5/2_	Pd_5/2_	Pd	Au		
Au/VGCNF	83.4	–	16.6	98.8	–	1.2	–	84.1
Pd/VGCNF	85.7	14.3	–	98.15	1.85	–	335.0	–
Pd_1_Au_1_/VGCNF	80.8	8.8	335.1	335.1	1.2	0.8	335.1	83.9

### Electrochemical Analysis for Glycerol Oxidation in Base Solution

**Fig. 5:**
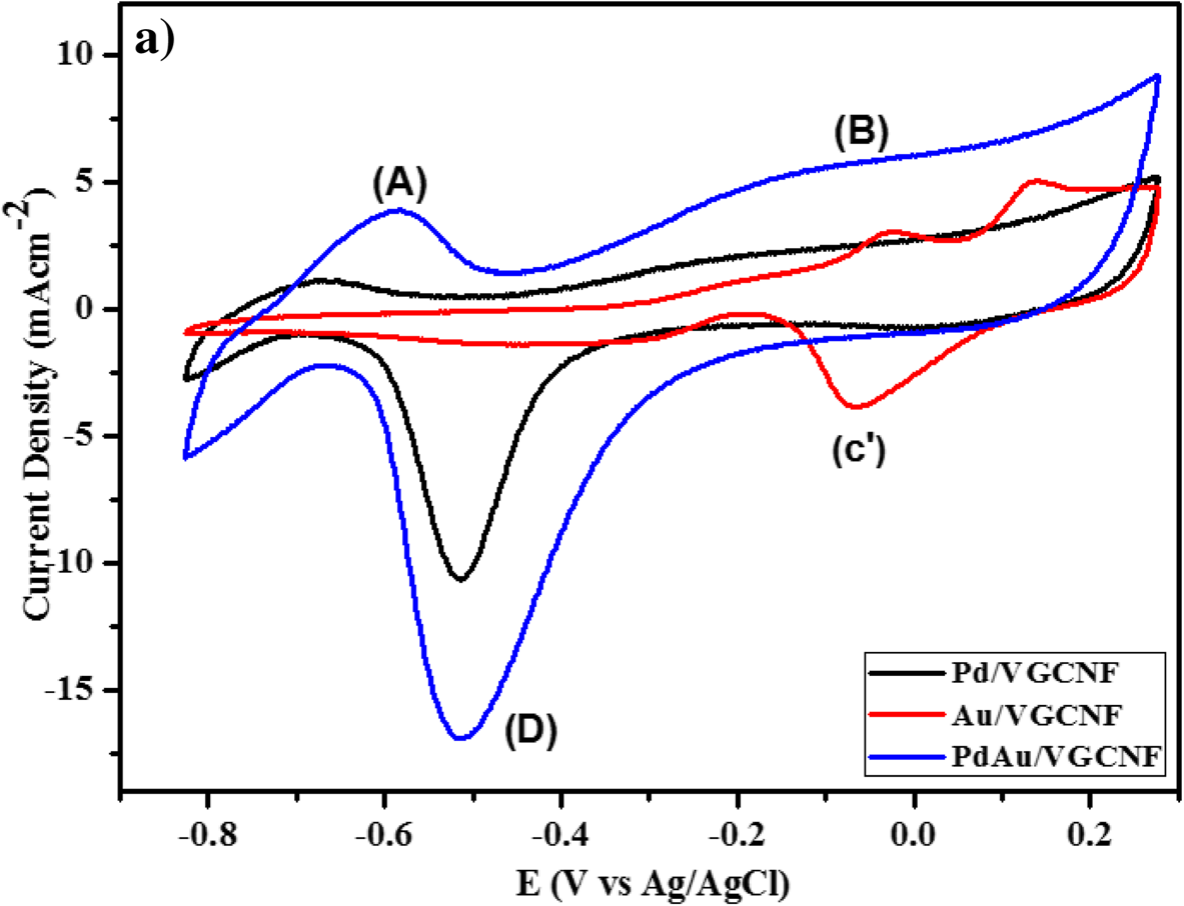
Cyclic voltammogram for Pd/VGCNF, Au/VGCNF, and Pd_1_Au_1_/VGCNF in 0.5 M glycerol

The CV scan of the catalyst was carried out after the catalyst loading on the glassy carbon electrode for approximately 24 h. The loading time indirectly influences the current density and peak potential (Ep). As the loading time increases, more time is required for the total amount of hydrogen evolved from the sample. The current density tremendously increases from the onset potential related to hydrogen desorption. The typical cyclic voltammetry curve in basic solution is generally used for the determination of hydrogen and oxygen adsorption on Pd/VGCNF, Au/VGCNF, and Pd_1_Au_1_/VGCNF in 0.5 M NaOH solution as depicted in Fig. 5. Region A in the potential range from − 0.8 to − 0.5 V can be assigned solely to adsorption and desorption of the H^+^ ion on electrocatalyst surface area. However, the adsorption and desorption peaks cannot be clearly distinguished. This is possibly caused by the blocking of certain electrocatalyst active sites upon the incorporation of the second metal [33]. In region B, in the intermediate potential between − 0.4 V and − 0.2 V, the catalyst behaves as an ideally polarizable catalyst (double layer region) where the surface has free-adsorbed H^+^ and OH^−^. Meanwhile, the formation of oxides occurs in the forward scan at the higher potential more than − 0.2 V between 0.1 and 0.3 V. The oxide reduction peak at − 0.34 V is also observed during the reverse scan in region C [33]. Meanwhile, Au/VGCNF electrocatalyst shows a slightly different voltammetry standard curve, where region A is the large capacitive current associated with the double layer region and is a typical electrochemical behavior of the Au catalyst followed by area B where the absorption of hydroxyl OH^−^ species and oxide formation is at higher potential above − 0.2 V vs Ag/AgCl, while area C is observed as a reduction region of Au oxides.

**Fig. 6:**
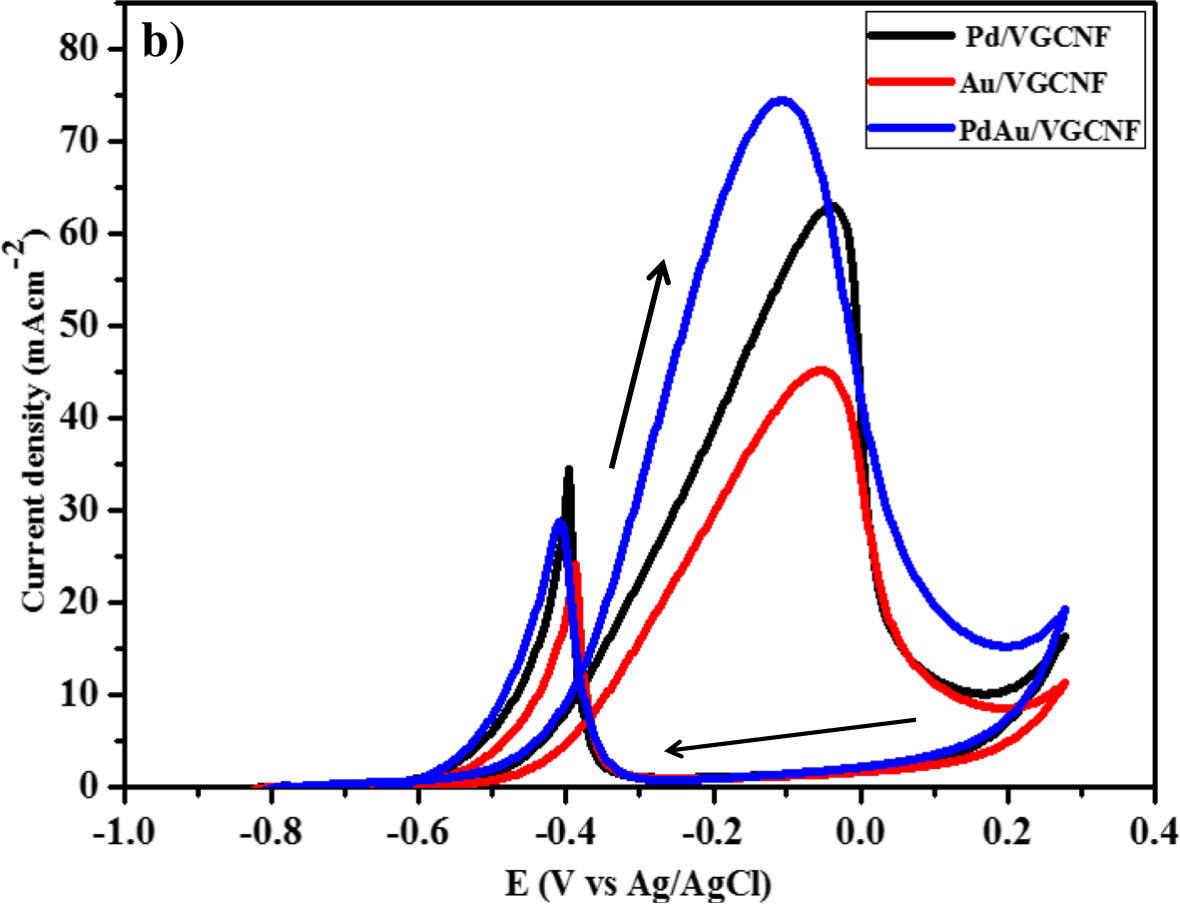
Cyclic voltammogram for Pd/VGCNF, Au/VGCNF, and PdAu/VGCNF in 0.5 M glycerol + 0.5 M NaOH

Figure 6 shows the anodic peak current density for Au/VGCNF, Pd/VGCNF, and Pd_1_Au_1_/VGCNF electrocatalysts for the oxidation of glycerol in alkaline medium. The performance in cyclic voltammetry curve of the prepared electrocatalysts towards the oxidation of the glycerol is seen in terms of four parameters: (1) onset oxidation potential, (2) forward anodic peak potential, (3) forward anodic peak current, and (4) CO tolerance as can be seen in Table 1 are investigated. The oxidation peak in forward scan can be attributed to the oxidation of freshly chemisorbed species from glycerol adsorption, and the oxidation peak in negative scan means the removal of carbonaceous species incompletely oxidized the forward scan. As a result, the ratio of the forward current density peak (*I*_f_) to the backward current density peak (*I*_b_) reflects the tolerance ability to carbonaceous species accumulation. A high ratio *I*_f_/*I*_b_ indicates efficient oxidation of glycerol during the forward scan and little accumulation of carbonaceous residues. In terms of peak current density, Pd_1_Au_1_/VGCNF provided the best performance compared to Pd/VGCNF and Au/VGCNF. Besides that, Pd_1_Au_1_/VGCNF also shows the lowest onset potential (− 0.75 V vs. > − 0.60 for glycerol). The worst results were obtained with methanol, especially in terms of current density and oxidation overpotential. After a detailed perusal of the literature and estimating that the metal catalyst loading is very low (0.1 mg cm^−2^), one may readily realize that Pd_1_Au_1_/VGCNF can be classified among the most active electrocatalysts ever reported for the oxidation of glycerol in half-cells. The current density for glycerol oxidation with electrocatalysts Pd/C, Pt/C, and Au/C is significantly higher compared to Zhang et al. [26] due to higher catalyst loading and temperature dependence.

In Table 3, it exhibits that the bimetallic PdAu/VGCNF has high electrocatalytic activity towards the glycerol oxidation with respect to monometallic electrocatalyst. The presence of a second metal which is Au leads to a slight increase of the activity between 0.45 and 0.7 V versus Ag/AgCl, as higher current densities are achieved. Looking at CV curve (Fig. 6) recorded at Pd/VGCNF and Au/VGCNF electrocatalysts, both materials presented almost the same onset potential for the glycerol oxidation close to − 0.4 V vs Ag/AgCl. The onset potential for PdAu/VGCNF shifted towards a lower value due to the bifunctional mechanism which gives the catalytic improvement. In agreement with the bifunctional mechanism, Au is an oxyphilic material which has oxygen adsorbing character which promotes the chemisorbed species and assists the electrooxidation of glycerol leading to the decrease of the onset potential of glycerol electrooxidation and enhanced catalytic activity [34]. Besides that, bimetallic electrocatalysts PdAu/VGCNF also examined excellent CO tolerance, when the ratio *I*_f_/*I*_b_ is superior than others, which reveals the higher catalytic efficiency for direct oxidation during the forward scan.

**Table 3:** Electrochemical analysis evaluations by cyclic voltammetry study

Electrocatalysts	E_on_ (V vs Ag/AgCl)	I_f_ (mA cm^−2^)	I_r_ (mA cm^−2^)	EASA (m^2^/g)	I_f_/I_r_
Au/VGCNF	− 0.4991	63.04	34.43	16.62	1.83
Pd/VGNCF	− 0.4771	45.17	24.22	14.51	1.86
Pd_1_Au_1_/VGNCF	− 0.5211	74.45	28.76	28.34	2.63

### Tafel Analysis

**Fig. 7:**
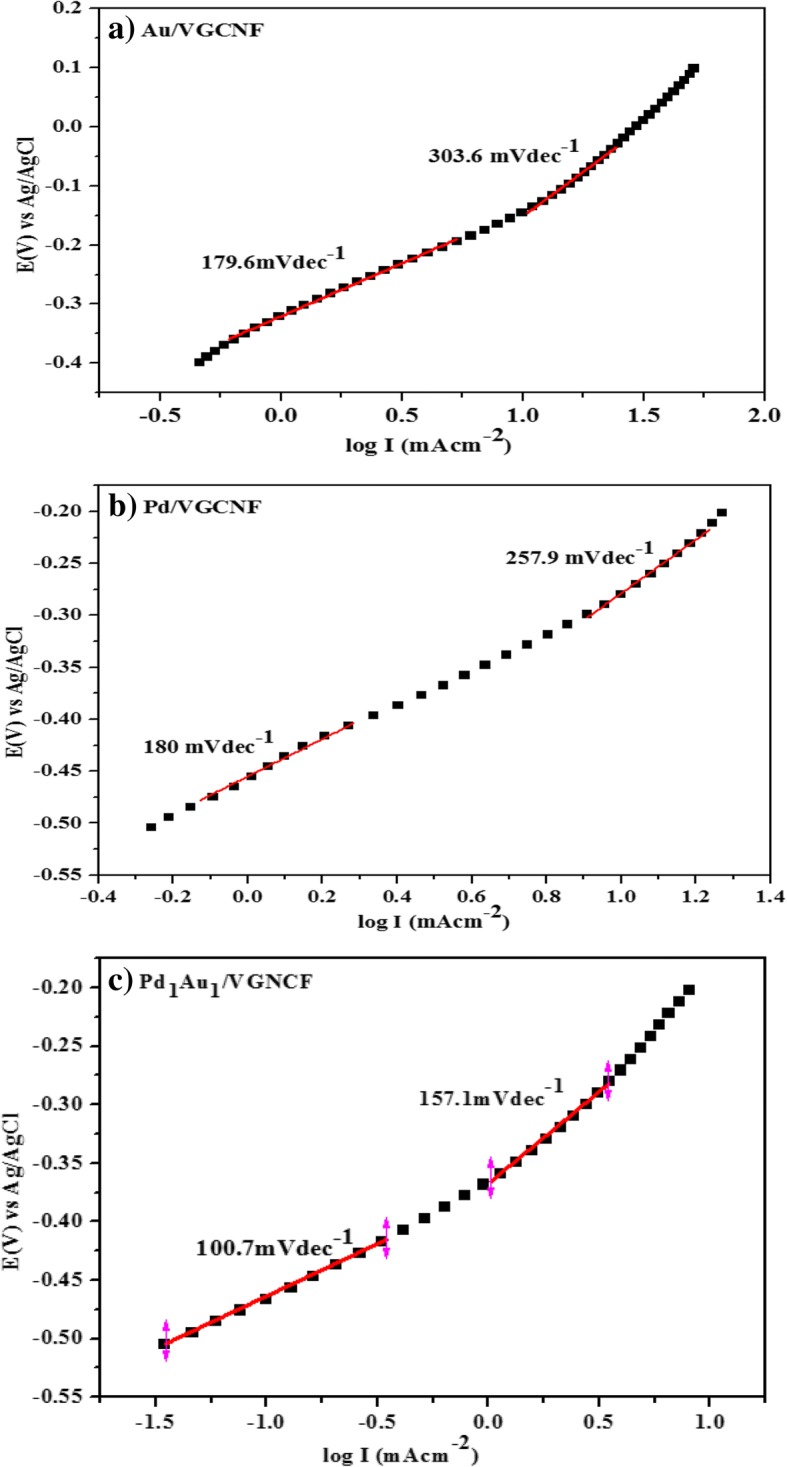
Tafel slope for Au/VGCNF, Pd/VGCNF, and Pd_1_Au_1_/VGCNF electrocatalysts in solution (0.5 M NaOH + 0.5 M Glycerol) at scanning rate 50 mVs^− 1^

Figure 7a–c shows a Tafel plot of all the electrocatalysts tested in the reaction of the glycerol oxidation. Each Tafel plot shows two linear areas with an increase in Tafel slope. The Tafel slope is one of the kinetic parameters associated with the determination of a reaction that occurs on the surface of the electrode. At low oxidation potential (E_v_), the Tafel slope is low which indicates the kinetic transfer charge is fast during the reaction of the glycerol oxidation. At higher oxidation potential (E_v_), the value of the second Tafel slope is greater which indicates that the electrocatalytic reaction mechanism changes. The change in Tafel slope can be attributed to the kinetic rate of the charge transfer in the reaction decrease due to the presence of intermediate species which can poison the surface of the electrocatalysts (Kang et al. [35]).

Table 4 lists the values of Tafel slope obtained on low and high potential oxidation. The Tafel slope for Pd_1_Au_1_/VGCNF electrocatalyst is lower than Pd/VGCNF and Au/VGCNF indicating that the charge transfer kinetic on bimetallic electrocatalyst is faster than single metal electrocatalyst for the glycerol oxidation reaction. Once the potential oxidation (E_v_) is increased, the Tafel slope also increased from low to high. This indicates that the reaction mechanism of the glycerol oxidation changes over the first one. Similar to the phenomenon occurring in other alcohol oxidation such as methanol [36] and ethanol [37], the maximum mass transfer of glycerol occurs in high potential oxidation (E_v_) which makes the hydroxyl adsorption and formed the oxide layer at electrocatalysts surface.

**Table 4:** Two different Tafel slope values on different potential oxidation

Electrocatalysts	Tafel slope (1) mVdec^−1^	Tafel slope (2) mVdec^−1^
Au/VGCNF	179.6	303.6
Pd/VGCNF	180.0	257.9
Pd_1_Au_1_/VGCNF	100.7	157.1

### Performance of Au/VGCNF, Pd/VGCNF, and PdAu/VGCNF in Direct Glycerol Fuel Cell

#### Electrochemical I-V Characterization

**Fig. 8:**
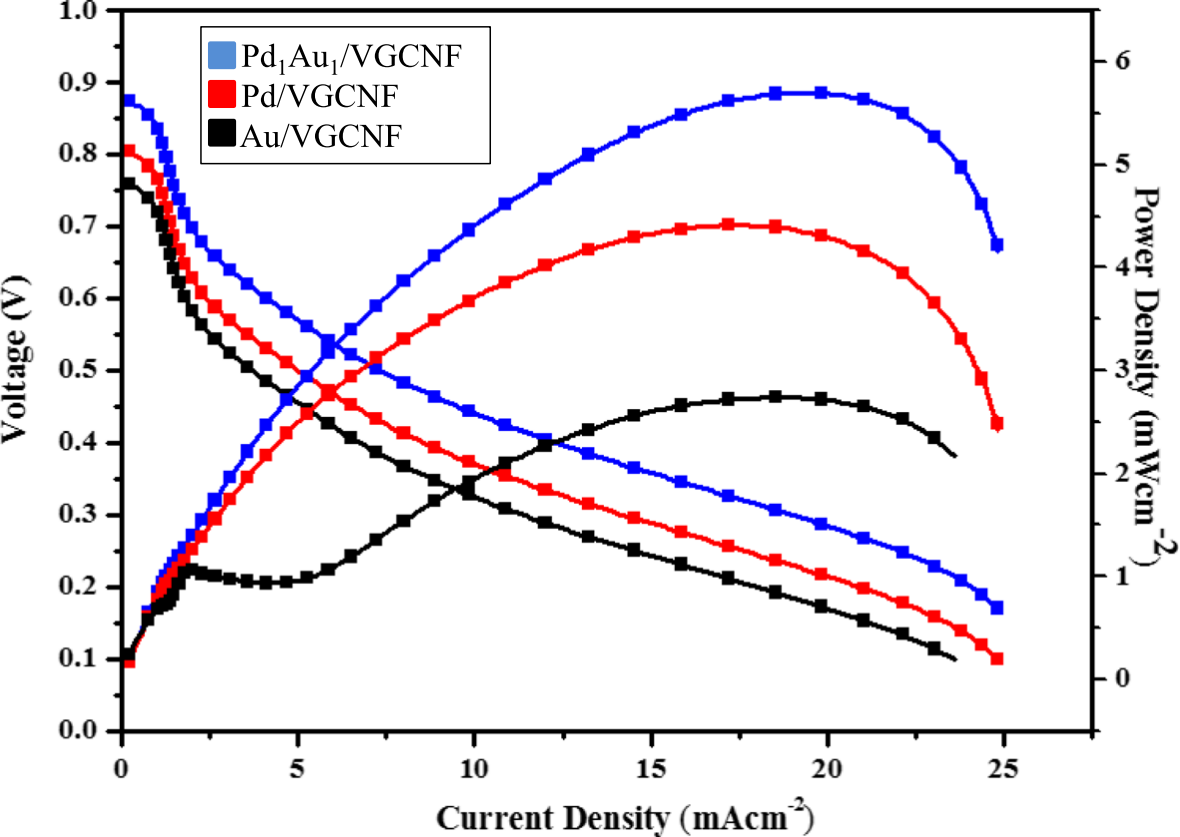
Polarization and power density curves for direct glycerol fuel cell (DGFCs) using PdAu/VGCNF at 25 ^°^C

Reliability of any synthesized electrocatalyst can generally be defined as the probability of the electrocatalyst to carry out its desired activity, which in this study is the catalysis for direct glycerol fuel cell applications. Although the electrocatalyst was analyzed earlier using a half-cell and showed good performance, the application of the electrocatalyst in direct glycerol fuel cells does not show outstanding performance. Figure 8 illustrates the cell polarization and power density curves of DGFC with FAA-3 (FuMA-Tech) membrane which show the passive DGFC single cell test results for Au/VGCNF, Pd/VGCNF, and Pd_1_Au_1_/VGCNF. From the DGFC performance test, results clearly show that the OCV and power density of Pd_1_Au_1_/VGCNF are higher compared to Pd/VGCNF and Au/VGCNF electrocatalysts. Table 5 lists the performance of all electrocatalysts in DGFC application in this study. In terms of electrocatalysts prices, roughly the price for Pd_1_Au_1_/VGCNF is USD 0.26/mg cheaper than the price for black Pd commercial which is USD 0.41/mg.

**Table 5:** Single cell output power density in mW cm^−2^ at different NaOH concentration solutions

Electrocatalysts (anode)	Types of membrane	Metal loading (mg cm^−2^)	OCV (V)	Power density (mW cm^−2^)
Pd_1_Au_1_/VGCNF	Fumatech	2.0	0.87	5.74
Pd /VGCNF	Fumatech	2.0	0.81	4.41
Au/VGCNF	Fumatech	2.0	0.76	2.74

Passive-performance alkaline fuel cell may not be able to challenge the performance of the active fuel cell. One of the main problems for the passive alkaline fuel cell is related to the liquid electrolyte such as NaOH or KOH. This is due to the very spontaneous reaction with the NaOH electrolyte solution which is very sensitive to the presence of CO_2_ from the air [38]. The use of air instead of oxygen will expose the hydroxyl ions to CO_2_ contained in the air and will lead to the formation of Na_2_CO_3_ or K_2_CO_3._ The presence of carbonates will indirectly reduce the amount of OH ions in the anode and thereby reduce the ionic conductivity [39]. Too much carbonate will block the pores of the gas diffusion layer and can severely degrade the performance of the single cell [31–33] .

#### Electrochemical Impedance Spectroscopy

**Fig. 9:**
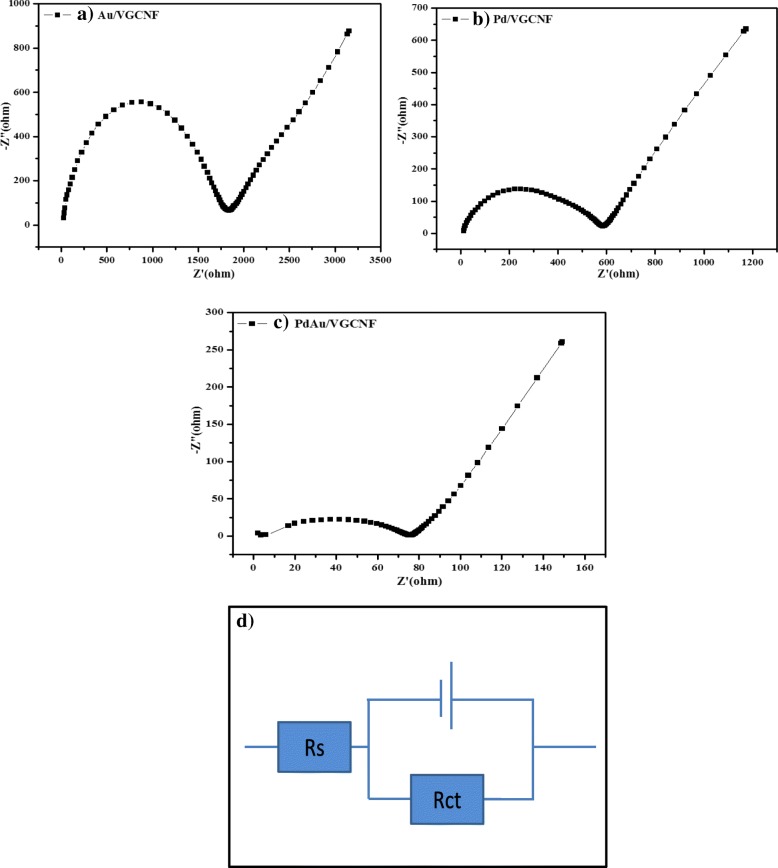
Nyquist plots of electrochemistry impedance spectra for glycerol oxidation reaction of **a** Au/VGCNF, **b** Pd/VGCNF, **c** PdAu/VGCNF, and **d** circuit.

The performance of the direct glycerol fuel cell assessed by polarization curves does not reflect and give enough information about the condition of the inner component. Thus, electrochemical impedance spectroscopy (EIS) has to be carried out together with polarization curve measurements to evaluate the overall kinetic performance such as distinguishing the influences (voltage drop caused by charger transfer, membrane or mass transfer resistance) of the different processes (kinetic, ohmic, or mass transport dominated) in the whole electronic system [40]. Figure 9a–c shows the Nyquist plots which are one typical curve of electrochemical impedance spectroscopy (EIS) for three different types of electrocatalysts. Generally, the diameter of the semicircle arc is to measure the charge–transfer resistance for glycerol oxidation reactions. Apparently, the DIA in Fig. 9 showed the order as follows: Au/VGCNF > Pd/VGCNF > PdAu/VGCNF. This suggests that PdAu/VGCNF has the greatest charge transport performance, which was consistent with the abovementioned outstandingly excellent electrochemical catalytic activity. Besides that the equivalent circuit which was fit to EIS spectra shown in Fig. 9d in which Rs represents the solution resistance and Rct represents the charge–transfer resistance for the process of glycerol electrocatalytic oxidation. Overall, the Rct value for PdAu/VGCNF is much smaller compared to other electrocatalyst which reveals that the electron transfer for glycerol oxidation on PdAu/VGCNF is much easier, which could enhance the electrocatalytic activity of the electrocatalyst for glycerol oxidation.

Since there is no difference in the MEA and cell assembly between the two cells, the lower resistance of PdAu/VGCNF cell can be attributed to the catalyst layer. In the PdAu/VGCNF-coated layer, small catalyst particle size can allow sufficient humidifying water to be held in the electrode and the uniform particle distribution can prevent the agglomeration effect, which may lead to the increasing boundary resistance. Furthermore, the small particle and uniform distribution can decrease the migration path of ions, which can further reduce the distributed resistance. The high-frequency EIS results provide the information that in the real fuel cell operation, the smaller cathode catalyst particle size will not only facilitate the ORR kinetics, but also reduce the performance loss that is due to the ohmic and contact resistances.

### MEA Morphology Analysis

Basically, the low performance of the fuel cell may be due to the inherent properties of the materials used in fuel cell construction, defects in the materials, and the assembly process of the fuel cell. Below, we further discuss the potential effects that give rise to the lower performance of direct glycerol fuel cell.

#### Microporous Layer (MPL) Cracking

**Fig. 10:**
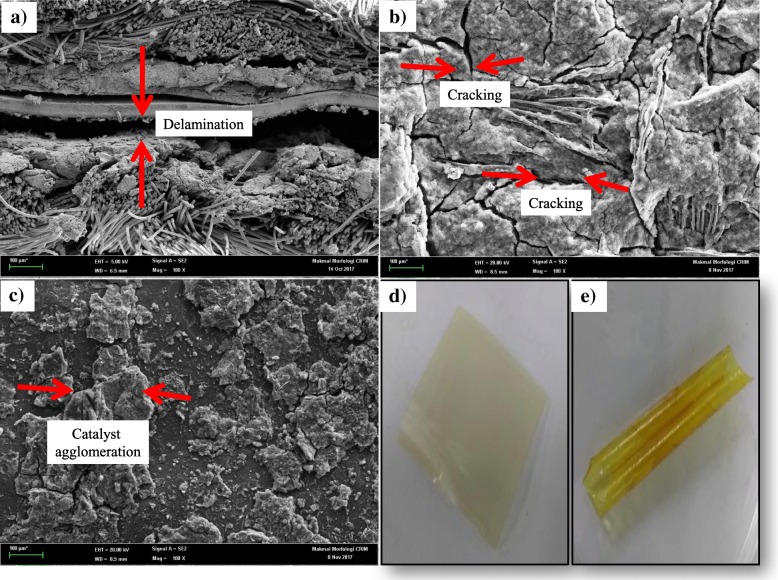
**a** Delamination. **b** MPL cracking. **c** Catalyst agglomeration

To justify the low performance of direct glycerol fuel cell, the morphology of some MEA were evaluated by SEM after the performance evaluation in the fuel cell. Figure 10a shows the typical SEM surface image for the microporous layer (MPL) consisting of the carbon black layer and the catalyst layer. Cracks were observed on the catalyst surface. The formation of wrinkles and cracks that occur in the MPL is likely due to a chemical reaction that cannot be controlled during the operation of single cell. This reaction probably causes dimensional changes in the polymer electrolyte membrane such as volume expansion and contraction that occur due to the hydrothermal stresses during the fuel cell operation [41]. Catalyst drying process occurs irregularly, causing cracks in the catalyst layer [42]. The fuel cell still can be operated in the presence of catalyst cracking. However, this indirectly degrades the fuel cell performance.

#### Delamination

The SEM image presented in Fig. 10b clearly shows an obvious gap between the membrane and the electrodes after the operation of the direct glycerol fuel cell. This could be caused by the thermal expansion mismatch between the membrane and electrodes occurring during the operation of the fuel cell. In addition, this may also occur during the manufacturing process of the MEA such as owing to a too high temperature used during the catalyst layer drying process, which may form a vapor which will be trapped and thereby create an area where the adhesion between the two layers is poor [43]. Nevertheless, the electrolyte in the catalyst ink is expected to act as a binder during the lamination process. However, this may not occur in practice because the manual catalyst casting will lead to wrinkles on the electrode resulting in the existence of voids between the layers and subsequent degradation in the performance. Another reason for the gap in the interface may be the fact that mechanical hot pressing is difficult to ensure a fully intimate membrane–electrode interface. This is another factor which affects the fuel cell performance. This condition will increase the contact resistance and will require a longer time for the proton to travel to the catalyst sites and the existence of dead zone can occur which indirectly led to the loss of catalyst activity.

#### Orientation of Catalyst Layer

The catalyst layer was cast by manual casting. This method is difficult to control, and it has the potential to cause the agglomeration of undispersed catalyst powder [44, 45]. Figure 10c shows the areas corresponding to the agglomeration of nanoparticles on the MPL layer. This can create contact resistance over the entire MEA and therefore adversely impact the porosity or proton conductivity.

#### AEM Stability

The anion exchange membrane (AEM) shows some technological and scientific limitation. AEM suffers from a poor chemical stability in alkaline media arising from the hydroxide attack on the cationic group and resulting in the decreased anionic conductivity [46]. The AEM with OH^−^ ion is less stable because AEM is easily converted to the less conductive ${\mathrm{CO}}_3^{2-}$ and even less conductive ${\mathrm{HCO}}_3^{-}$ forms when exposed to air even for short time. Figure 10d, e shows the comparison between before and after the treatment with alkali, indicating that AEM faced the problem of high swelling with the increased hydration [47]. This is a challenge in the handling of AEM. AEM is used to overcome the Nafion membrane degradation problem. However, AEM has a tendency to be hydrocarbon or aromatic based which gives rise to poor oxidation stabilities [41].

## Conclusion

The palladium-based electrocatalysts supported with VGCNF was synthesized and characterized in this work as effective electrocatalysts for the electrooxidation in the alkaline environment of glycerol as renewable resources. The well-dispersed Au, Pd, and Pd_1_Au_1_ verified by XRD, TEM, and HTREM on VGCNF help to reduce the precious metal loading and served as a high reaction zone. The oxidation of glycerol has been primarily investigated by a variety of electrochemical techniques. The results obtained have highlighted the excellent electrocatalytic activity of bimetallic electrocatalyst which is PdAu/VGCNF as high as 74.45 mA cm^−2^ followed by Au/VGCNF and Pd/VGCNF. The high electrocatalytic activity of Pd_1_Au_1_/VGCNF could be related to the high dispersion of the metal particles and to the intrinsic properties of the VGCNF. In addition, the performance of passive DGFC applying MEA with the synthesized Pd_1_Au_1_/VGCNF was evaluated. The peak power density of the passive DMAFC was 3.9 mW cm^−2^, for glycerol concentration of 2.0 M and NaOH concentration of 5.0 M. The EIS showed that PdAu/VGCNF shows smaller arc which indicates a smaller kinetic resistance in comparison with two other electrocatalysts. The extensive evaluation of single cell manufacturing indicates that power output performance is also affected by GDL morphological defects, MEA manufacturing, and AEM stabilities. Therefore, it is apparent that more research on alkaline fuel cell is needed to address the problem relevant for improving the reliability during the long-term operation of DMFCs.

## Abbreviations

AEM: Anion exchange membrane
Au: Aurum
BET: Brunauer, Emmett, and Teller
CV: Cyclic voltammetry
EDX: Energy-dispersive X-ray
FESEM: Field emission scanning electron microscopy
HAuCl_4_·3H_2_O: Gold(III) chloride trihydrate
MPL: Microporous layer
N_2_: Nitrogen
Pd: Palladium
PdCl_2_: Palladium chloride
PTFE: Polytetrafluoroethylene
VGCNF: Vapor-grown carbon nanofiber
XPS: X-ray photoelectron spectroscopy
XRD: X-ray diffraction
